# Immunomodulatory role of Interleukin-33 in large vessel vasculitis

**DOI:** 10.1038/s41598-020-63042-3

**Published:** 2020-04-14

**Authors:** Anne-Claire Desbois, Patrice Cacoub, Aurélie S. Leroyer, Edwige Tellier, Marlène Garrido, Anna Maciejewski-Duval, Cloé Comarmond, Stéphane Barete, Michel Arock, Patrick Bruneval, Jean-Marie Launay, Pierre Fouret, Ulrich Blank, Michelle Rosenzwajg, David Klatzmann, Mohamed Jarraya, Philippe Cluzel, Fabien Koskas, Gilles Kaplanski, David Saadoun

**Affiliations:** 1 Sorbonne Universités, UPMC Univ Paris 06, INSERM, UMR S 959, Immunology-Immunopathology- Immunotherapy (I3), F-75005 Paris, France; 2 Biotherapy (CIC-BTi) and Inflammation-Immunopathology-Biotherapy Department (DHU i2B), Hôpital Pitié-Salpêtrière, AP-HP, F-75651 Paris, France; 3 AP-HP, Groupe Hospitalier Pitié-Salpêtrière, Department of Internal Medicine and Clinical Immunology, F-75013 Paris, France; 4 Centre national de références Maladies Autoimmunes et systémiques rares et Maladies Autoinflammatoires rares, Paris, France; 5 Aix-Marseille Univ; INSERM, Vascular Research Center of Marseille, UMR-S 1076, 13385 Marseille, France; 6 Laboratoire de biotechnologies et pharmacologie génétique appliquée, CNRS UMR 8147, ENS - Ecole normale supérieure de Cachan, Cachan, France; 7 AP-HP, Hôpital Pitié-Salpétrière, Laboratoire d’Hématologie Biologique, Paris, France; 8 Laboratoire d’anatomopathologie, Hôpital Européen Georges Pompidou, Paris, France; 9 INSERM, UMR-S 942, F-75010 Paris, France; 10 Laboratoire d’anatomopathologie; Groupe Hospitalier Pitié-Salpétrière, Paris, France; 11 Inserm U1149, CNRS ERL8252, Faculté de Médecine Site X. Bichat, Paris, France; 12 Banque des tissus Humains, Hôpital saint Louis, Paris, France; 13 Service de radiologie vasculaire, Groupe Hospitalier Pitié-Salpétrière, Paris, France; 14 Service de Chirurgie vasculaire, Groupe Hospitalier Pitié-Salpétrière, Paris, France; 15 APHM, CHU Conception, Service de Médecine Interne, Marseille, France

**Keywords:** Cell signalling, Interleukins

## Abstract

The mechanisms regulating inflammation in large vessels vasculitis (LVV) are poorly understood. Interleukin 33 (IL-33) has been shown to license innate and adaptive immunity by enhancing Th2 cytokines production. We aimed to examine the role of IL-33 in the immunomodulation of T cell activation in LVV. T cell homeostasis and cytokines production were determined in peripheral blood from 52 patients with giant cell arteritis (GCA) and 50 healthy donors (HD), using Luminex assay, flow cytometry, quantitative RT-PCR and by immunofluorescence analysis in inflammatory aorta lesions. We found increased level of IL-33 and its receptor ST2/IL-1R4 in the serum of patient with LVV. Endothelial cells were the main source of IL-33, whereas Th2 cells, Tregs and mast cells (MC) express ST2 in LVV vessels. IL-33 had a direct immunomodulatory impact by increasing Th2 and Tregs. IL-33 and MC further enhanced Th2 and regulatory responses by inducing a 6.1 fold increased proportion of Tregs (p = 0.008). Stimulation of MC by IL-33 increased indoleamine 2 3-dioxygenase (IDO) activity and IL-2 secretion. IL-33 mRNA expression was significantly correlated with the expression of IL-10 and TGF-β within aorta inflammatory lesions. To conclude, our findings suggest that IL-33 may exert a critical immunoregulatory role in promoting Tregs and Th2 cells in LVV.

## Introduction

Large vessel vasculitis (LVV) mainly include giant cell arteritis (GCA). LVV may lead to segmental stenosis, occlusion, dilatation and/or aneurysm formation in the aorta and/or its main branches^1^. Histological lesions are characterized by an inflammatory infiltrate located in the inner part of aortic wall (*media*) associated with a fragmentation of the internal elastic lamina and hyperplasia of *vaso vasori*. LVV are driven by common immune mechanisms in the activation and regulation of CD4 T cells. Indeed, vascular lesion formation is mediated by inadequate immune response, characterized by *in situ* activation of CD4 T cells. Weyand *et al*., have shown the deficiency of the programmed cell death protein-1 immune checkpoint in GCA promoting CD4+ T cells activation^1^. Apart from the strength of the antigen/T-cell receptor (TCR) signal, the microenvironment is also critical in T-cell activation and polarisation. Previous studies have provided evidence that LVV inflammation was driven by Th1 and Th17 cells in the peripheral blood and inflamed tissues^2–4^. They also demonstrated a quantitative deficiency of regulatory T cells (Tregs)^5^. However, the mechanisms involved in regulation of CD4 T cell homeostasis in LVV are poorly understood and need to be further studied in order to highlight potential therapeutic targets.

Ciccia *et al*. have reported overexpression of IL-33 within inflammatory lesions of temporal arteries in GCA patients^6^. A meta-analysis has found an association of GCA and an IL-33 genetic variant^7^. Interleukin 33 (IL-33), a recently identified cytokine belonging to the IL-1 family^8^, is known to be implicated in the regulation of immune response in several human diseases^9–12^. IL-33 is constitutively expressed in the nucleus of epithelial, endothelial cells and also fibroblasts and has been shown to license innate and adaptive immunity by enhancing Th2 cytokines production. IL-33 is secreted secondary to cell apoptosis and necrosis. IL-33 binds to a heterodimeric receptor called ST2/IL-1R4, expressed mainly on Th2 cells, Tregs, mast cells, eosinophils, basophils, Group 2 Innate Lymphoid Cells (ILC2s) and Natural Killer (NK) cells^13^. During inflammation, several mechanisms allow the regulation of IL-33 effects, such as sST2, a soluble form of ST2 acting as a decoy receptor that inhibits IL-33 signalling. Studies have demonstrated that IL-33 is a key regulator of the immune response, and is involved in many autoimmune and inflammatory disorders. However, its immune function in patients with LVV is largely unknown.

The present study examined the critical role of IL-33 to modulate T cell activation in LVV.

## Methods

### Patients

The study population consisted of 52 GCA patients (median [IQR] age at diagnosis: 74.7 [66.3; 83.2] years), fulfilling the international criteria for GCA, respectively^14,15^. Clinical characteristics of LVV patients are indicated in Table 1. For IL-33 measurement in sera, 50% and 24.5% of samples were performed in patients with active disease and without treatment, respectively. For functional experiments, PBMC and CD4+ T cells were obtained from patients who were untreated or receiving corticosteroids<10 mg/day. Blood samples from 50 age and sex-matched healthy donors (HD) were obtained from Etablissement Français du Sang (Hôpital Pitié-Salpêtrière) and were used as controls. Aorta biopsies were obtained from consecutive GCA patients undergoing surgical repair for aortic aneurysm or dissection. Three non-inflammatory aorta biopsies were provided by a tissue bank and obtained from vessels taken for transplant in mutlti-organ and tissue donors. Temporal arteries and aorta were obtained at diagnosis and some aortas specimens were collected during a vascular complication.

**Table 1:** Main demographical and clinical characteristics of LVV patients.

Parameters	GCA n-=52
Demographic features Median age [IQR]	74.7[66.3; 83.2]
Female gender	35 (67.3%)
Geographic origin	
Caucasian	50 (96.2%)
African	0 (0%)
North African	2 (3.8%)
Other	0 (0%)
**Clinical features**	
Stroke	7 (13.4%)
Aortic aneurysms	4 (7.7%)
Aortitis	14 (26.9%)
Ocular complications	5 (9.6%)

The study was approved by our institutional ethics review board (*Comité de Protection des Personnes-Ile-de-France-VI*) and was performed according to the Helsinki declaration. Donors gave informed consent.

### Analysis of cytokine production

Levels of IL-33 *in sera* were performed using enzyme-linked immunosorbent assay (ELISA, R&D systems) in LVV patients and HD. ST2 level *in sera* was performed using enzyme-linked immunosorbent assay (ELISA, R&D systems) in GCA patients and HD. Quantitative determination of Th1 (IFNγ), Th17 (IL-17), Th2 (IL-4, IL-5, IL-13) cytokines, IL-10 and IL-6, was performed in culture supernatant using Human Cytokine 25-Plex (Invitrogen, France) in accordance with the manufacturer protocol.

For intracellular staining, peripheral blood mononuclear cells (PBMCs) were stimulated for 4 hours with 0,05 µg/mL Phorbol 12-myristate 13-acetate (PMA) and 1 mM (1 µg/mL) ionomycin (Sigma-Aldrich) in the presence of brefeldin A (BD Pharmingen) and were stained with the following conjugated monoclonal antibodies, at predetermined optimal dilutions, for 15 minutes at room temperature: CD3-APC-Alexa Fluor 750, CD4-ECD, CD8-APC-Alexa Fluor 700, (Beckman Coulter). Intracellular detection of IL-4-PE (BD, France), IFNγ-FITC (Miltenyi Biotec), TNFα-PE (Miltenyi Biotec), IL17-eFluor 660 (ebioscience), IL-10-APC (BD, France) was performed on fixed and permeabilized cells using appropriate buffer (BD Pharmingen).

For analysis of regulatory T cells (Tregs), intranuclear detection of FOXP3-AF647 (Beckman Coulter), with CD3-FITC (Beckman Coulter), CD8-KO (Beckman Coulter), CD4-PB (Beckamn Coulter), CD127-PC7 (Beckamn-Coulter), CD25-PE (BD) was performed using PerFIX-nc Kit (Beckman Coulter). Data were acquired using a Navios flow cytometer and analyzed with the Kaluza analysis software (Beckman Coulter).

For all analyses, the gating strategy consisted in excluding debris and dead cells, then selecting CD3+ T cells among lymphocytes, and thereafter studying markers of interest among those CD3+ CD4+ T cells.

### T cell differentiation with IL-33

Peripheral blood mononuclear cells (PBMCs) of LVV patients were cultured in RPMI-1640 medium supplemented with 10% Fetal Bovine Serum (FBS) and 2% penicillin-streptomycin (1 × 10^6^ cells/ml) and stimulated in 48-well plates coated with anti-CD3/CD28 monoclonal antibodies with or without 10 ng/mL human recombinant IL-33 (eBiosciences). After 3 and 5 days of culture, culture supernatants were harvested and immediately frozen. Quantitative determination of IL-4, IL-10, IL-5, IL-13 and IFN_γ_, IL17 in culture supernatant was performed using Human Milliplex® kit (Merck Millipore, France) in accordance with the manufacturer protocol. Intracellular expression of Th1 and Th2 cytokines was determined by flow cytometry (Navios, Beckman Coulter). The proportion of CD4+ CD25High CD127Low FOXP3+ cells among TCD4+ cells was assessed by flow cytometry.

In order to investigate the impact of mast cells on Tregs in presence of IL-33, CD4+ T cells (1 × 10^6^) of LVV patients were purified from PBMC according the instructions of the manufacturer (Stemcell ®) and were co-cultured in 24-well plates coated with anti-CD3/CD28 monoclonal antibodies for 4 days with or without human ROSA^KITWT^ ^16^ mast cells (2.5 × 10^5^) in the presence or the absence of recombinant IL-33. The proportion of FOXP3+ CD25High CD4+ cells in each condition was assessed by flow cytometry as described previously.

### Assessment of indoleamine-2, 3-deoxigenase (IDO) activity

The kynurenin to tryptophan ratio was used as a surrogate indicator of IDO activity. Levels of tryptophan and kynurenin were analyzed by isocratic liquid chromatography with coulometric detection^17^.

### Immunofluorescence analysis

Detection of IL-33^+^, Von Willebrand Factor, ST2/IL-1R4^+^, CD3^+^, FOXP3+, Tryptase^+^, IL-10^+^, VEGF (Vascular Endothelial Growth Factor) and IL-4^+^ cells was performed on fixed, paraffin-embedded samples from 5 consecutive temporal arteries and 13 consecutive aorta of GCA patients with active inflammatory arterial lesions and 5 temporal arteries of controls and 3 non-inflammatory aorta. After dewaxing in baths of xylene and ethanol, slides were submitted to antigen retrieval by heating in citrate buffer pH 6.0. Before incubation with primary antibodies, Fc receptor was blocked with normal goat serum 5%. Slides were incubated over night with monoclonal mouse anti-human CD3 (dilution 1:50, Abcam) rabbit polyclonal anti Von Willebrand Factor (dilution 1:250, Dako Cytomation), mouse monoclonal anti-IL-33 (dilution 1:1000, Enzo Life Sciences), rabbit polyclonal anti-ST2 (dilution 1:100, Sigma-Aldrich), rabbit polyclonal anti-IL-4 (dilution 1:50,Abcam), rabbit polyclonal anti IL-10 (dilution 1:25, Abcam), rat monoclonal anti-FoxP3 (1:100, eBioscience), mouse anti-tryptase (1:400, DAKO) or with isotype control: polyclonal Rabbit IgG or monoclonal mouse IgG (Abcam) or monoclonal rat IgG (eBioscience). Slides were then incubated for 2 hours at room temperature with Cy3-conjugated goat anti-mouse (working dilution 1:1000, Jackson Immunoresearch) and Alexa 488 donkey anti- rabbit (working dilution 1:1000, Life Technologies), mounted in Mowiol, and evaluated under fluorescence microscopy.

### Gene expression quantification at the mRNA level in aortic tissues

Quantification of mRNA expression was performed on paraffin-embedded aorta samples from GCA (n = 20) patients and non-inflammatory aorta controls (n = 3). ST2, IL-1β, IL-10, TNFα, IL-6, IL-8, IFNγ, IL-4, GATA-3, FOX-P3, T-BET, ROR-γt, CD-45 genes were analyzed. The theoretical and practical aspects of real-time quantitative reverse transcriptase-polymerase chain reaction (RT-PCR) using the ABI Prism 7900 Sequence Detection System (PerkinElmer Applied Biosystems, Foster City, CA) have been described in detail elsewhere (13). Briefly, total FFPE RNA was extracted using the High Pure FFPET RNA Isolation Kit (Roche) following the supplier’s protocol and reverse transcribed before real-time PCR amplification. Gene mRNA expression levels were quantified by using real time RT-PCR. Quantitative values were obtained from the cycle number (*C*_t_ value) at which the increase in the fluorescence signal associated with exponential growth of PCR products started to be detected by the laser detector of the ABI Prism 7900 Sequence Detection System (Perkin-Elmer Applied Biosystems), using PE Biosystems analysis software according to the manufacturer’s manuals. The precise amount of total RNA added to each reaction mix (based on optical density) and its quality (i.e., lack of extensive degradation) are both difficult to assess. We therefore also quantified transcripts of the *RPLP0* gene (Genbank accession: NM_001002) encoding the ribosomal protein subunit P0 as an endogenous RNA control and normalized each sample on the basis of its *RPLP0* content. Results, expressed as *N*-fold differences in target gene expression relative to the *RPLP0* gene and termed “*N*_target_,” were determined as *N*_target_ = 2^Δ*C*t-*sample*^, where the Δ*C*_t_ value of the sample was determined by subtracting the average *C*_t_ value of the target gene from the average *C*_t_ value of the *RPLP0* gene. The *N*_target_ values of the samples were subsequently normalized to obtain a ‘basal mRNA level’ (smallest amount of mRNA quantifiable (Ct=35)) equal to 1. The primers for all genes were chosen with the assistance of the Oligo 6.0 program (National Biosciences). We scanned the dbEST and nr databases to confirm the total gene specificity of the nucleotide sequences chosen for the primers and the absence of SNPs. To avoid amplification of contaminating gDNA, 1 of the 2 primers was placed at the junction between 2 exons or on 2 different exons. Agarose gel electrophoresis was used to verify the specificity of PCR amplicons.

### Culture of temporal arteries

Temporal arteries of GCA patients (n = 8) and controls (n = 5) were cultured for 24 hours without or with 10 ng/ml recombinant human IL-33 for 24 hours. Temporal arteries were then incubated for 24 h in RNAlater at +4 °C and then frozen at −80 °C. Culture supernatants were collected and immediately frozen at −80 °C. The determination of cytokines dosages in supernatants was performed using Milliplex® kit (Merck Millipore, France) in accordance with the manufacturer protocol. Quantification of IL-1β, IL-10, TNFα, IL-6, IL-8, IFNγ, IL-4, CD-45 mRNA was also performed. Temporal arteries were lyzed and homogenized. Total RNA was extracted by TRIzol reagent (Invitrogen) and reverse-transcribed using SuperScript VILO cDNA Synthesis Kit (Invitrogen) both according to the manufacturer’s instruction. Gene expression was determined by real-time PCR. Each cDNA sample was amplified in triplicate using SYBR Green (Applied Biosystems) on 7500 FAST Real-time PCR System (Applied Biosystems). The thermal cycling conditions comprised an initial denaturation step at 95 °C for 10 minutes, followed by 40 cycles at 95 °C for 15 seconds and 65 °C for 1 minute. Quantitative values were obtained from the threshold cycle (Ct) number at which the increase in the signal associated with exponential growth of PCR products began to be detected. *GADPH* gene was used as an endogenous control and each sample was normalized on the basis of its *GADPH* content and the expression of CD45. The primers were designed to span introns and are listed in supplementary Table.

### Statistics

Continuous variables are presented with the median and range or with the mean ± SEM. Categorical variables are presented with counts and proportions. Statistical comparisons were performed by using the Mann-Whitney test for quantitative unpaired data, and the Wilcoxon matched pairs test for quantitative paired data. All statistical tests were two-tailed with a significance level of 0.05. Statistical significance was evaluated using GraphPad Prism version 5.00 for Windows (GraphPad Software, San Diego, CA, USA).

## Results

### Expression of Th2 and regulatory cytokines in blood and inflamed vessels in LVV

IL-33 is known to be implicated in the regulation of immune response by enhancing Th2 cytokines production and Tregs. To study the effects of IL-33 on CD4+ T cells homeostasis in LVV, we first evaluated if Th2 and regulatory responses were present in blood and inflamed vessels in LVV patients besides the secretion of Th1 and Th17 cytokines, previously shown in LVV (Fig. S1).

**Figure 1:**
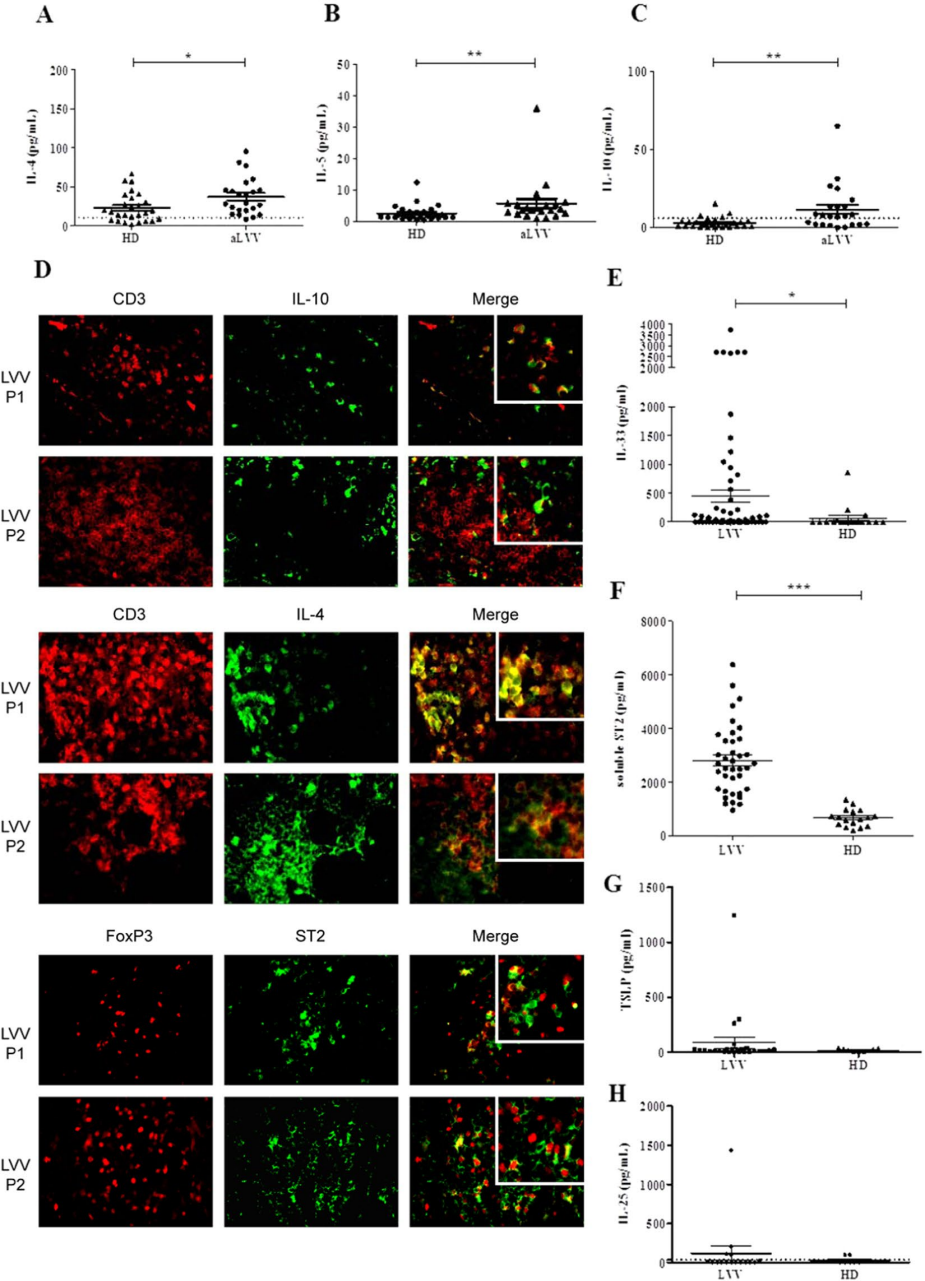
Expression of Th2 and anti-inflammatory cytokines in LVV. (**A–C**) PBMC of active LVV patients (aLVV) (n = 23) and HD (n = 27) were stimulated for 4 hours with PMA-ionomycine. Expression levels of interleukin 4 (IL-4), interleukin 5 (IL-5) and interleukin 10 (IL-10) measured in supernatant are represented. The levels of IL-4, IL-5 and IL-10 are higher in LVV patients as compared to HD [p = 0.01; p = 0.002 and p = 0.003, respectively]. We used a Mann Whitney test. These data are shown as the mean ± SEM. (**D**) Immunofluorescence analysis of inflammatory lesions from LVV patients reveals the expression of Th2 (here IL-4) and IL-10 cytokines partially by CD3 positive cells. Expression of ST2 by Treg (defined as FOXP3 cells) by immunofluorescence analysis. (**E**) Expression level of IL-33 in LVV (n = 44) patients serum was higher compared to HD (n = 18), *P < 0.05. (**F**) Mean level of soluble ST2 was increased in LVV patient sera (n = 38) as compared to HD (n = 17), ***P < 0.001. (**G**) Level of TSLP in LVV (n = 16) patients serum compared to HD (n = 9). (**H**) Level of IL-25 in LVV (n = 16) patients serum compared to HD (n = 9).

We first evaluated the level of Th2 and regulatory (IL-10) cytokines in culture supernatant of PBMC after 4 hours of PMA-ionomycine stimulation. Comparisons of cytokines between LVV patients and HD are presented in Fig. 1A–C. Active LVV patients had significantly increased median levels of IL-4 (p = 0.01), IL-5 (p = 0.002) and IL-10 (p = 0.003) as compared to HD. Consistently, the quantitative analysis of mRNA within inflammatory lesions of LVV aorta revealed an overexpression of GATA 3 (11 ± 17), as compared to controls with non-inflammatory aorta aneurysms (p = 0.006) (Supplementary Data S2). Immunofluorescence analyses of aorta tissue specimens from patients with LVV also revealed an expression of IL-4 and IL-10, partially by T cells (Figs. 1D and S3). Within inflammatory lesions, immunofluorescence analysis of aorta in patients with LVV also revealed that Tregs (defined as positive FOXP3 cells) expressed ST2/IL-1R4 (Figs. 1D and S3). Consistently, the quantitative analysis of mRNA within inflammatory lesions of LVV aorta revealed an overexpression of FOXP3 (44.2 ± 55.2, p = 0.005), as compared to controls with non-inflammatory aorta aneurysms (**data not shown**).

We next assessed in LVV patients the expression of IL-33 and other cytokines known to promote Th2 polarisation (i.e IL-25 and TSLP (Thymic stromal lymphopoietin). We found overexpression of IL-33 in LVV relative to HD (Fig. 1E). A significant increased level of IL-33 was observed in sera of LVV patients as compared to HD (449.4 (±894.9) vs 67.7 (±204.6) pg/mL, p = 0.002) (Fig. 1E). Consistently, mean level of IL-33 receptor (soluble ST2) was increased in sera of LVV patients as compared to HD (n = 17) [2808.7 (±1284.7) and 677.7 (±322) in HD, p < 0.0001] (Fig. 1F). In contrast, levels of IL-25 and of TSLP were not different in LVV and in HD (Fig. 1G,H).

### IL-33 and its receptor ST2 are overexpressed in pathological vessels of LVV patients

**Figure 2:**
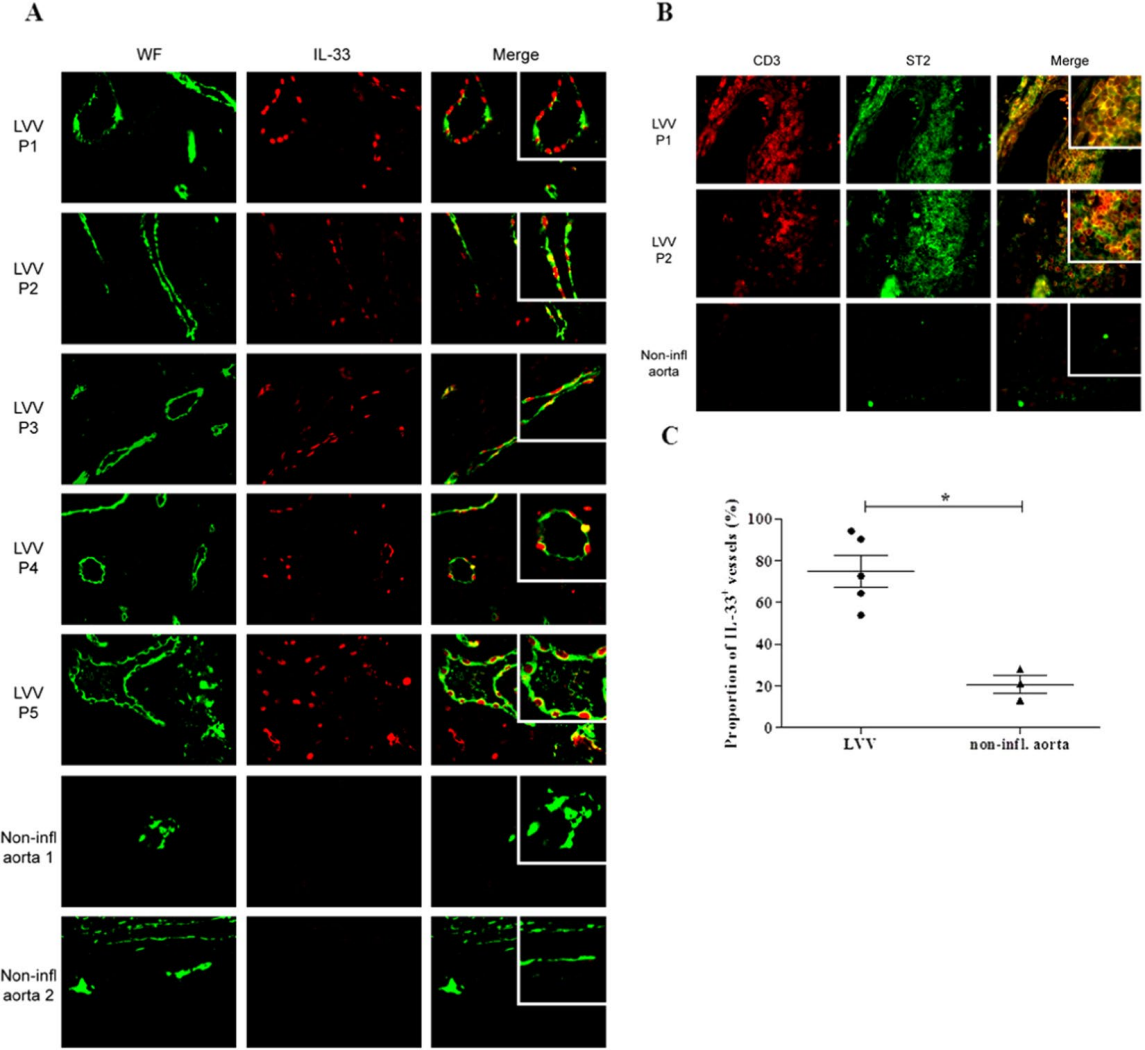
IL-33 and its receptor ST2 were overexpressed in LVV. (**A**) Immunofluorescence analyses of aorta tissue specimens from LVV patients revealed that IL-33 was mainly expressed within adventitial vessels and co-localized with endothelial cells with positive von willebrand factor (WF) staining. Immunofluorescence analysis of non-inflammatory aorta did not reveal IL-33 positive staining. (**B**) Expression of ST2 was mainly observed within the inflammatory infiltrates in aorta tissue specimen. Immunofluorescence analyses of one non-inflammatory aorta show vessels (WF positive staining) without positive IL-33 staining and absence of ST2 staining. Immunofluorescence analysis of non-inflammatory aorta did not reveal ST2 positive staining. (**C**) The proportion of vessels with positive IL-33 staining was higher in aorta from LVV patients (n = 5) as compared to non-inflammatory controls (n = 3), *P < 0.05. These data are shown as the mean ± SEM.

We next studied the expression of IL-33 in LVV inflammatory vessels and controls. Immunofluorescence analyses of temporal arteries and aorta tissue specimens from LVV patients and controls were used to investigate the expression pattern of IL-33 and ST2/IL-1R4. IL-33 was mainly expressed within adventitial vessels in co-localization with endothelial cells [i.e. von willebrand factor (WF)] and in a lesser extent within few inflammatory cells in aorta (Fig. 2A) and temporal arteries. Expression of ST2/IL-1R4 was observed within the inflammatory infiltrates of aorta **(**Fig. 2B**)** and temporal arteries of LVV patients. The proportion of IL-33 positive vessels was higher in aorta of LVV (n = 5) patients as compared to controls (p = 0.036) **(**Fig. 2C**)**.

### IL-33 predominantly induces a Th2 and regulatory immune responses in LVV

**Figure 3:**
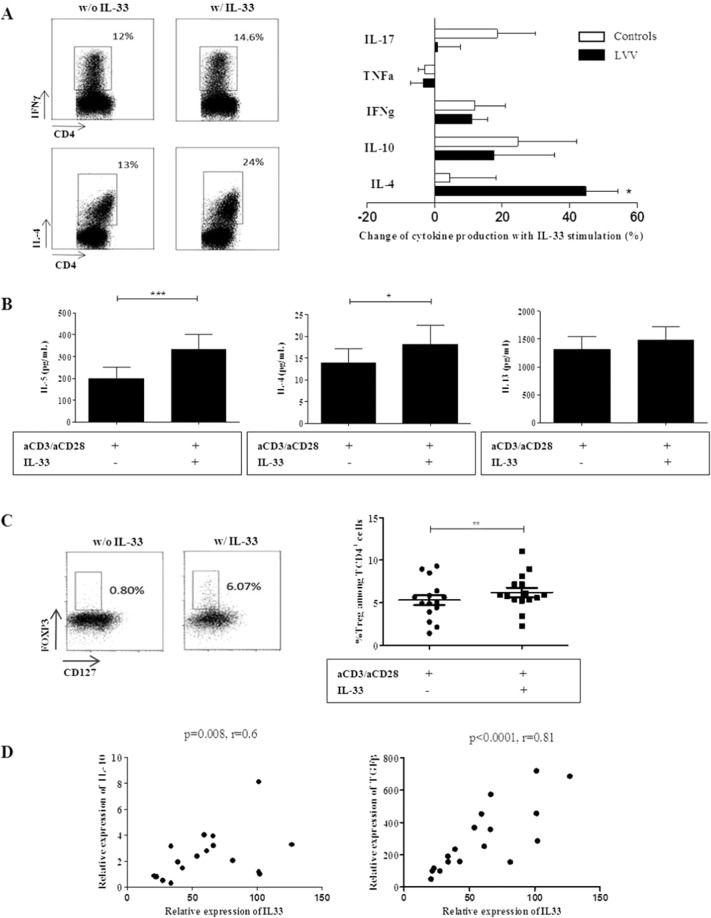
Il-33 predominantly induced a Th2 and regulatory immune response in LVV. (**A**) Freshly isolated PBMC from LVV patients (with corticosteroids<15 mg/day) were cultured with anti-CD3/CD28 with or without IL-33 stimulation for 5 days. The secretion of Th1 and Th2 cytokines was assessed by flow cytometry. Left panel: Dot plots representing IFNγ and IL-4 secreting CD4+ T cells with or without IL-33 stimulation. Right panel: Changes of cytokine production in PBMC stimulated with IL-33 compared to PBMC not stimulated. The proportion of IL4-secreting CD4^+^ T cells was increased with IL-33 stimulation (p = 0.01) in LVV patients (n = 13) but not in HD (n = 6). These data are shown as the mean ± SEM. These results are from 13 independent experiments. (**B**) After 5 days of culture, quantitative determination of cytokines was performed in culture supernatants (n = 12) of LVV PBMC. IL-33 stimulation led to a significant increase of IL-5 and IL-4 secretion, *p < 0.05, ***P < 0.001. (**C**) Left panel: Dot plots representing CD25^hi^FOXP3^+^ and CD127^low^FOXP3^+^ CD4^+^ cells are shown. On the left, PBMC of one LVV patient are cultured for 5 days without IL-33. The frequency of CD127^low^FOXP3^+^ CD4^+^ cells is shown. On the right, PBMC of the same LVV patient were cultured for 5 days with IL-33. Right panel: Corresponding results of 15 LVV patients. The frequency of Tregs was increased in stimulated PBMC with IL-33 as compared to those without IL-33. These results are from 4 independent experiments. These data are shown as the mean ± SEM. **P < 0.01. The statistical test used was a Wilcoxon matched pair test. (**D**) We next assessed by quantitative PCR the expression of IL-33, ST2 and Th1 and Th2 cytokines within LVV aortic lesions (n = 18). Relative expression of IL-33 mRNA was significantly correlated with the expression of IL-10 mRNA [r = 0.6 (p = 0.008)]. Relative expression of IL-33 mRNA was significantly correlated with the expression of TGF-b mRNA [r = 0.8 (p < 0.0001)].

Through ST2 binding, IL-33 is known to be able to induce helper T cells, mast cells and eosinophils to produce type 2 cytokines in allergy. We next attempted to determine whether IL-33 overexpression was accompanied by an increase in Th2 cytokines in LVV. Freshly isolated PBMC from LVV patients (with corticosteroids<15 mg/day) and controls were cultured with anti-CD3/CD28 with or without IL-33 stimulation for 5 days. The secretion of cytokines was evaluated by flow cytometry (Figs. 3A, S4) and by quantitative determination in culture supernatants (Fig. 3B**)** after 5 days of PBMC culture with anti-CD3/CD28 monoclonal antibodies.

The proportion of CD4^+^ cells secreting IL-4 was increased after IL-33 stimulation [9.6 (±11.7) % vs 7.06 (± 9.1) %, p = 0.006] in LVV patients but not in HD **(**Fig. 3A**)**. IL-33 stimulation led to an increase of IL-5, IL-4 and IL-13 production in culture supernatants of PBMC after 5 days of culture with anti-CD3/CD28 monoclonal antibodies [332.7 (±237.2) pg/ml with IL-33 vs. 200.1 (±176.1) pg/ml without IL-33, p = 0.005 and 18.1 (±15.4) pg/ml with IL-33 vs 13.9 (±11.4) pg/ml without IL-33, p = 0.02 and 1479.5.6 (±669.9) pg/ml with IL-33 vs 1317.6 (±644.9) pg/ml without IL-33, p = 0.07, respectively], but not of IFN_γ_, IL-17 and IL-10 (Fig. 3A,B).

Since regulatory T cells (Treg) can also express ST2/IL-1R4, we next studied the impact of IL-33 on the proportion of Tregs. We have shown that IL-33 induced an increased proportion of Tregs after 5 days of PBMC culture [5.3% without IL-33 vs 6.2% with IL-33, (p = 0.001)] **(**Figs. 3C, S5**)**. In addition, Tregs expanded by IL-33 were functional (i.e. assessed by their ability to inhibit effector T cells proliferation, Fig. S6).

We next studied the correlation between Th2 and regulatory cytokines and IL-33 expression in LVV arteries in order to study the *in vivo* effects of IL-33. We assessed by quantitative PCR (qPCR) the expression of IL-33, ST2, Th1 and Th2 cytokines within inflammatory aortic lesions of 18 LVV patients who underwent a surgical repair for an aortic aneurysm/dissection. Relative expression of IL-33 mRNA was significantly correlated with the expression of IL-10 and TGF-β mRNA [r = 0.6 (p = 0.008) and r = 0.8 (p < 0.0001), respectively] **(**Fig. 3D**)**. The relative expression of IL-33 was not correlated with the expression of T-Bet, RORγt, GATA3 and IFNγ. The relative expression of ST2 was correlated with those of FOXP3 and GATA-3 [r = 0.6 (p = 0.0035) and r = 0.6 (p = 0.009), respectively] (**data not shown**). Altogether, these data suggest a significant correlation between IL33/ST2 axis and regulatory immune response *in vivo*.

To further precise the impact of IL-33 on the immune response within inflammatory lesions, we assessed the secretion of cytokines secreted by GCA temporal arteries (n = 8) stimulated or not by IL-33. IL-33 stimulation tended to be associated with increased production of IL-10 by temporal arteries measured by both quantitative determination of cytokines in culture supernatants [51.4 (± 66.1) pg/ml vs 18.8 (±12.9) pg/ml] and qPCR [relative expression of 1.9 (±1.1)] from temporal arteries **(data not shown)**. IL-33 did not increase the production of IFNγ, nor IL-6 in culture supernatants of temporal arteries (**data not shown**).

### Synergic role of IL-33 and stimulated mast cells in immune regulation in LVV

**Figure 4:**
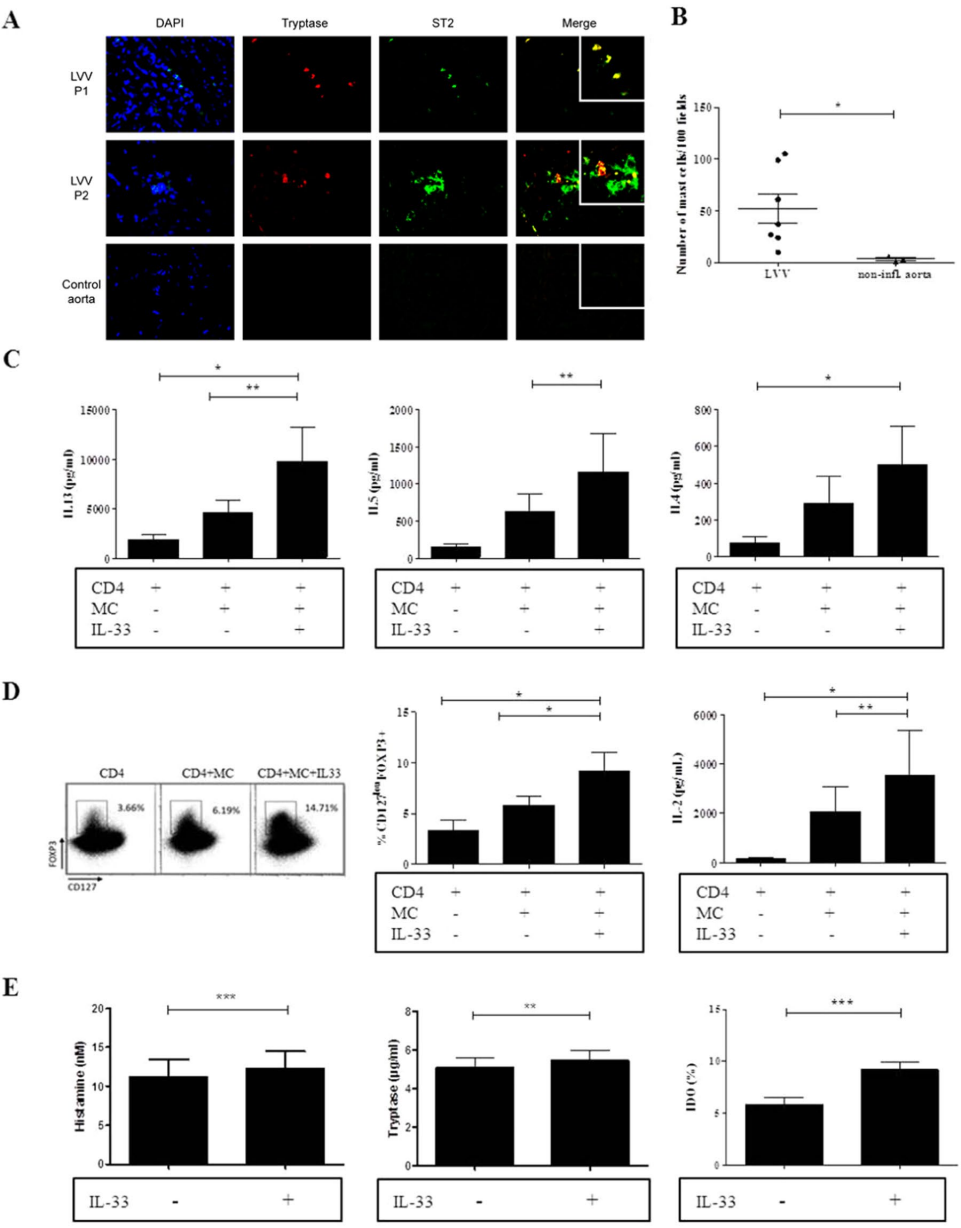
IL-33 enhanced a regulatory and Th2 immune response through MC. (**A**) Immunofluorescence staining of LVV inflammatory lesions of 2 LVV patients and 1 non-inflammatory aorta control for ST2 and MC. (**B**) The number of MC within aorta was higher in LVV patients (n = 7) than in non-inflammatory controls (n = 3) *P < 0.05. These data are shown as the mean ± SEM. (**C**) Quantitative determination of Th2 cytokines in culture supernatants (n = 8) of MC and CD4^+^ T cells. IL-33-stimulated MC led to a significant increase in IL-5, IL-13 and IL-4 secretion. *P < 0.05, **P < 0.01. These data are shown as the mean ± SEM. (**D**) Left panel: Dot plots representing the proportion of CD25^high^FOXP3^+^ and CD127^low^FOXP3^+^ CD4^+^ cells are shown. On the left: CD4^+^ T cells of one LVV patient are cultured for 5 days without IL-33. The proportion of CD127^low^FOXP3^+^ CD4^+^ cells is shown. On the middle: MC and stimulated CD4 T cells were cultured for 5 days without IL-33. On the right: MC and stimulated T CD4 cells were cultured for 5 days with IL-33. This patient is representative of the whole cohort (n = 8). Middle panel: The corresponding results of 8 LVV patients. MC alone and IL-33-stimulated MC promote the increase of Tregs frequency. *P < 0.05, **P < 0.01. These results are from 8 independent experiments. These data are shown as the mean ± SEM. Right panel: Quantitative determination of cytokines was performed in culture supernatants (n = 8). IL-33-stimulated MC led to a significant increase in IL-2 secretion. *P < 0.05, **P < 0.01. These data are shown as the mean ± SEM. (**E**) Left and middle panels: MC were incubated with LVV serum (n = 11) with or without IL-33 stimulation. IL-33 led to a discreet increase of MC degranulation (histamine and tryptase). Right panel: Indoleamine 2 3-dioxygenase (IDO) activity was dramatically increased in MC incubated with IL-33 as compared to those not stimulated with IL-33.**P < 0.01, ***P < 0.001. These data are shown as the mean ± SEM.

We have previously shown a direct role of IL-33 on T cells polarization. As mast cells (MC) represent one of the main targets of IL-33, we next studied whether MC stimulated by IL-33 could also enhance a regulatory immune response in patients with LVV. First, we demonstrated the presence of MC within inflammatory infiltrates of LVV aorta. Some mast cells in arterial wall had positive staining for IL-33 receptor (ST2/IL-1R4) (Fig. 4A). We have also shown by immunofluorescence an increased number of mast cells in LVV lesions (n = 7) within the aorta as compared to non-inflammatory controls [51.9 (± 37.6) in LVV vs 3.3 (± 3.5), p = 0.02] (Fig. 4B).

We first assessed the impact of IL-33 on mediators’ secretion of MC stimulated with patient serum. In these conditions, there was no impact of IL-33 on IL-4, IL-6, IL-8 and VEGF production (**data not shown**). Next, we have evaluated the impact of IL-33 stimulated- MC on Th2 polarization and on Tregs promotion. CD4^+^ cells of 8 LVV patients (treated with corticosteroids<10 mg/day) stimulated with αCD28/αCD3 were cultured alone or combined with MC with or without IL-33 for 4 days. IL-33-stimulated MC led to a significant increased secretion of IL-5 [144.7 (±121) in CD4 T cells vs 634.8 (±671.5) pg/ml in CD4 T cells and MC vs 1168.2 (±1461.5) pg/ml in CD4 T cells, MC and IL-33, p = 0.008], IL-13 [1895.4 (±1296.6) pg/ml in CD4 T cells vs 4643.2 (±3375.5) pg/ml in CD4T cells and MC vs 9800 (±9548.4) pg/ml in CD4 T cells, MC and IL-33, p = 0.008 and 0.03] and IL-4 [74.6 (±79.9) pg/ml in CD4 T cells vs 291.5 (±410.1) pg/ml in CD4 T cells and MC vs 499.5 (±585.9) pg/ml in CD4 T cells, MC and IL-33, p = 0.03] (Fig. 4C).

The proportion of Tregs was also determined in each previously described condition. We observed an increase in Treg number when stimulated CD4^+^ cells of LVV patients were cultured with MC as compared to the condition without MC. The addition of IL-33 to MC further increased the proportion of Treg as compared to the other conditions [median proportion of CD25^hi^FOXP3^+^CD4^+^T cells of 1.88 (0.34; 8.85) % in CD4^+^ T cells alone vs 5.57 (2.38; 7.79) % in CD4^+^ T cells plus MC and 11.02 (1.7; 14.84) % in CD4^+^ T cells plus MC with IL-33 (p = 0.02 and 0.008, respectively)]. Altogether, these results show that MC and IL-33 enhance a regulatory immune response (Figs. 4D and S7).

Finally, we studied by which mechanisms MC stimulated by IL-33 could enhance regulatory response. Quantitative determination of cytokines was also performed in coculture supernatants of MC and CD4^+^T cells (n = 8). IL-33-stimulated MC led to a significant increase of IL-2 secretion [136.4 (±167.4) pg/ml in CD4 T cells alone vs 2080 (±2808.9) pg/ml in CD4 T cells with MC vs 3555.4 (±5062) pg/ml in CD4 T cells, MC and IL-33, p = 0.008 and 0.03], which may explain the increase of Tregs by MC and IL-33 (Fig. 4D). IL-2 blocking led to a decrease in Treg frequency of 29% (n = 4).

IL-33 led to very discreet increase of degranulation by MC (Fig. 4E). However, we found an increased activity of indoleamine 2 3-dioxygenase (IDO) in MC incubated with LVV serum and IL-33 as compared to those not stimulated with IL-33 [9 (6.7; 15.4) % vs 5.8 (2.4; 10.8) %, p = 0.001]. The increased production of IDO, an important immunomodulatory mediator, also may explain the increase of Treg (Fig. 4E).

Together, the results indicate that IL-33 and MC further enhanced Th2 and regulatory responses by inducing a 6.1 fold increased proportion of Tregs (p = 0.008). Stimulation of MC by IL-33 increased indoleamine 2 3-dioxygenase (IDO) activity and IL-33 was associated with an increase in IL-2 secretion (by T cells and/or MC) which participates to production of Tregs.

## Discussion

The present study examined the critical role of IL-33 in regulating T cell activation in LVV. IL-33 has been previously found overexpressed of IL-33 in temporal arteries of GCA patients^6^. A meta-analysis has found an association of GCA and an IL-33 genetic variant^7^. However, the functional role of IL-33 in LVV patients is unknown. IL-33 is a key regulator of immune responses shown to license innate and adaptive immunity^18,19^.

Herein, we found overexpression of IL-33 and its receptor soluble ST2 in LVV patients. Endothelial cells were the main source of IL-33 in inflamed aorta. The analysis of cytokine production in LVV patients pointed out, besides Th1 and Th17 polarization, a secretion of Th2 cytokines increased in LVV patients compared to healthy controls. In addition, IL-4 and IL-10 were expressed within the inflammatory aorta of LVV patients. In hematopoietic cells, IL-33 is known to act primarily on immune cells associated with type 2 response^8,20^. Through ST2/IL-1R4 interactions, IL-33 induces helper T cells, mast cells and eosinophils to produce type 2 cytokines. Indeed, Th2 cells express constitutively high levels of ST2/IL-1R4. In Th2 GATA3^+^ cells, combined IL-33 and IL-2/STAT5 increase the expression of GATA-3 and hence promote Th2 program and enhance also the expression of ST2/IL-1R4^18^. IL-33 significantly modulates the immune response in favor of type 2 cytokine in LVV. They exert a synergistic effect on IL-5, IL-4 and IL-13 production in LVV patients whereas no significant change was observed for Th1 and Th17 cytokines. In addition, ST2 expression in inflamed aorta was correlated with GATA-3 and FOXP3. These findings are in line with data showing expression of ST2 by Th2 cells and Tregs^18^. IL-33 has been shown to exert protective effects in atherosclerosis mice through a switch from a pro-atherosclerotic Th1type to a protective Th2 type immune response. ^11^In cardiovascular disease models, IL-33 induction following vascular and cardiac stress was correlated with improved outcomes^3,11,12,15,27^. Taken together, IL-33 may tilt the Th1 and Th17 inflammatory responses observed in LVV patients.

Since Tregs expressed ST2/IL-1R4 in vessels of LVV, we next studied the impact of IL-33 on Tregs. We have shown that IL-33 expanded functional Tregs. Within inflammatory aorta lesions in LVV patients, the expression of IL-33 mRNA was correlated with those of TGF-β and IL-10 mRNA. Consistently, the addition of human rIL-33 to GCA temporal arteries was associated with an overexpression of IL-10. Besides Th2 polarization, IL-33 acts primarily on regulatory immune responses, through mast cells and Tregs^8,21^. Some Tregs express constitutively high levels of ST2/IL-1R4, IL-33 enhances FOXP3 expression and thereby Tregs program and also boosts the expression of ST2^18^. Tissue Tregs are highly suppressive and express IL-10. In inflammatory bowel diseases, ST2/IL-1R4 is preferentially expressed on colonic Tregs cells^22^ and IL-33 through ST2 promotes Treg function and adaptation to the inflammatory environment. IL-33 signalling enhances TGFb-mediated differentiation, accumulation and maintenance of Treg in inflamed tissue. Altogether, our data suggest a regulatory mechanism of the IL-33/Th2 axis that controls the Th1 and Th17 mediated inflammation. Consistent with the immunomodulatory role of IL-33 in LVV, IL-33 expression was observed at diagnosis in arteries during the acute phase of inflammation. However, we cannot exclude that IL-33 production also characterizes a “healing” stage of vasculitis occurring after the acute phase of inflammation. IL-33 reflects the plasticity of immune cells that secrete cytokines participating in immune regulation and tissue healing, according the phase of inflammation.

IL-33 and mast cells synergize the expansion of Tregs. Indeed, we showed that IL33 increases Tregs and that this effect is amplified by MC [about 6-fold increase with MC stimulated with IL-33 compared to CD4^+^ T cells alone]. Higher levels of IDO and of IL-2 were observed after stimulation of IL-33 stimulated mast cells. These two molecules are critical in the homeostasis of Tregs^23^ and may be key mediators of Tregs expansion. Although mast cells are known to contribute to inflammation in many conditions such as allergy or rheumatoid arthritis^24^, other reports suggest that they are also critical in enhancing a regulatory immune response^25–27^. Consistently, MC were found to be central for skin allograft tolerance in mice, via Treg-secreted IL-9 which leads to activation and recruitment of MC^25^. Rodrigues *et al*. recently found that dendritic cells with direct contact with MC expressed higher levels of PDL1, secreted higher levels of IDO and stimulated regulatory T lymphocytes producing IL-10 and TGF-β^26^. In a model of papain-induced allergy, MC stimulated with IL-33 have been shown to be crucial to suppress papain-induced inflammation by promoting regulatory T cells^28^. IL-33 was shown to both enhance airway eosinophilia and inflammation through ILC2 stimulation but also to lead to increased Treg number though MC and IL-2^28^. Thus, our results highlight immunomodulatory properties of mast cells in LVV, and suggest that mast cells might be a potential therapeutic target.

In conclusion, our results demonstrate an unrecognized link between an endogenous mediator of tissue damage and an anti-inflammatory pathway in LVV. We provide evidence that IL-33 may regulate inflammation in LVV through their action on Th2 and Tregs cells.

## Supplementary information

Supplementary Dataset 1.
