# Supplementary material for: Immunomodulatory role of Interleukin-33 in large vessel vasculitis
Source: Sci Rep. 2020 Apr 14;10:6405. doi: 10.1038/s41598-020-63042-3 (PMC7156501; doi:10.1038/s41598-020-63042-3)
Supplement: Supplementary file 1 — Supplementary Dataset 1. [file 41598_2020_63042_MOESM1_ESM.docx]

**Immunomodulatory role of Interleukin-33 in large vessel vasculitis**

**Anne-Claire Desbois^1,2,3^, Patrice Cacoub^1,2,3^, Aurélie Leroyer ^4^, Edwige Tellier ^4^, Marlène Garrido ^1,2^, Anna Maciejewski-Duval ^1,2^, Cloé Comarmond^1,2,3^, Stéphane Barete ^3^, Michel Arock ^5,6^ , Patrick Bruneval ^7^, Jean-Marie Launay ^8^, Pierre Fouret ^9^, Ulrich Blank ^10^, Michelle Rosenzwajg ^1,2,3^, David Klatzmann ^1,2,3^, Mohamed Jarraya ^11^, Philippe Cluzel ^12^, Fabien Koskas ^13^, Gilles Kaplanski ^3,14^, David Saadoun ^1,2,3^.**

**Figure S1: Th1 and Th17 polarisation in LVV.**

Figure S1A: Production of IFNg by PBMC of GCA patients compared to HD. ***P<0.001.


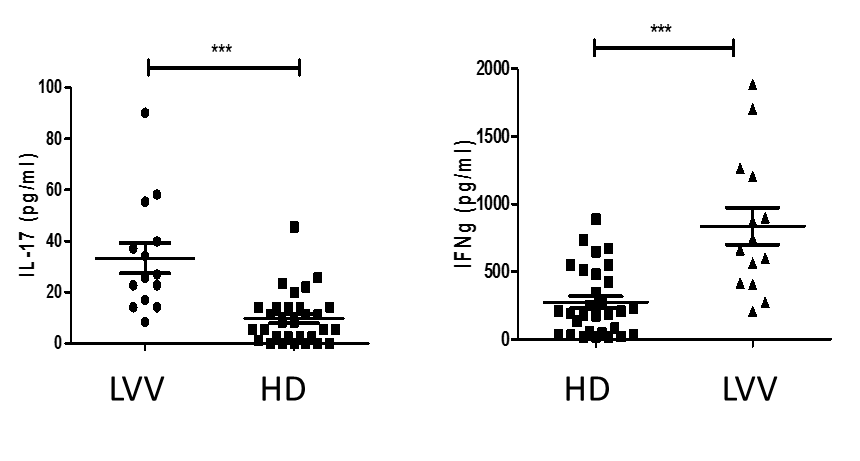


Figure S1B: Relative expression of IFNg mRNA and IL-33 mRNA compared to non- inflammatory aorta by qPCR.

**Figure S2:** **Relative expression of GATA in LVV aorta and controls**

**Figure S3**: Immunofluorescence analysis of IL-4, IL-10, CD3 and FOXP3 expression with DAPI staining in LVV arteries.

**
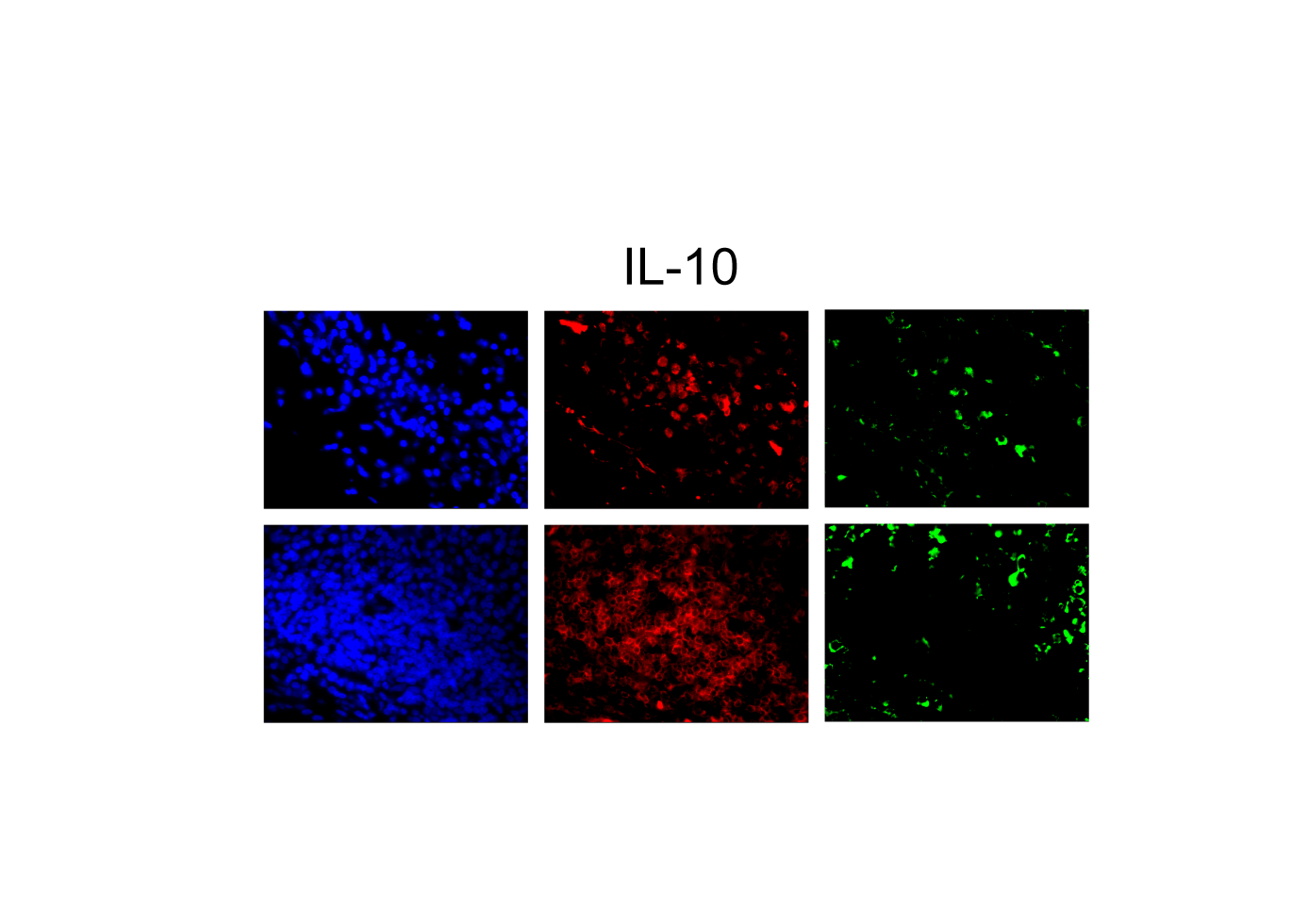
** DAPI CD3 IL-10

**LVV P1**

**LVV P2**

**
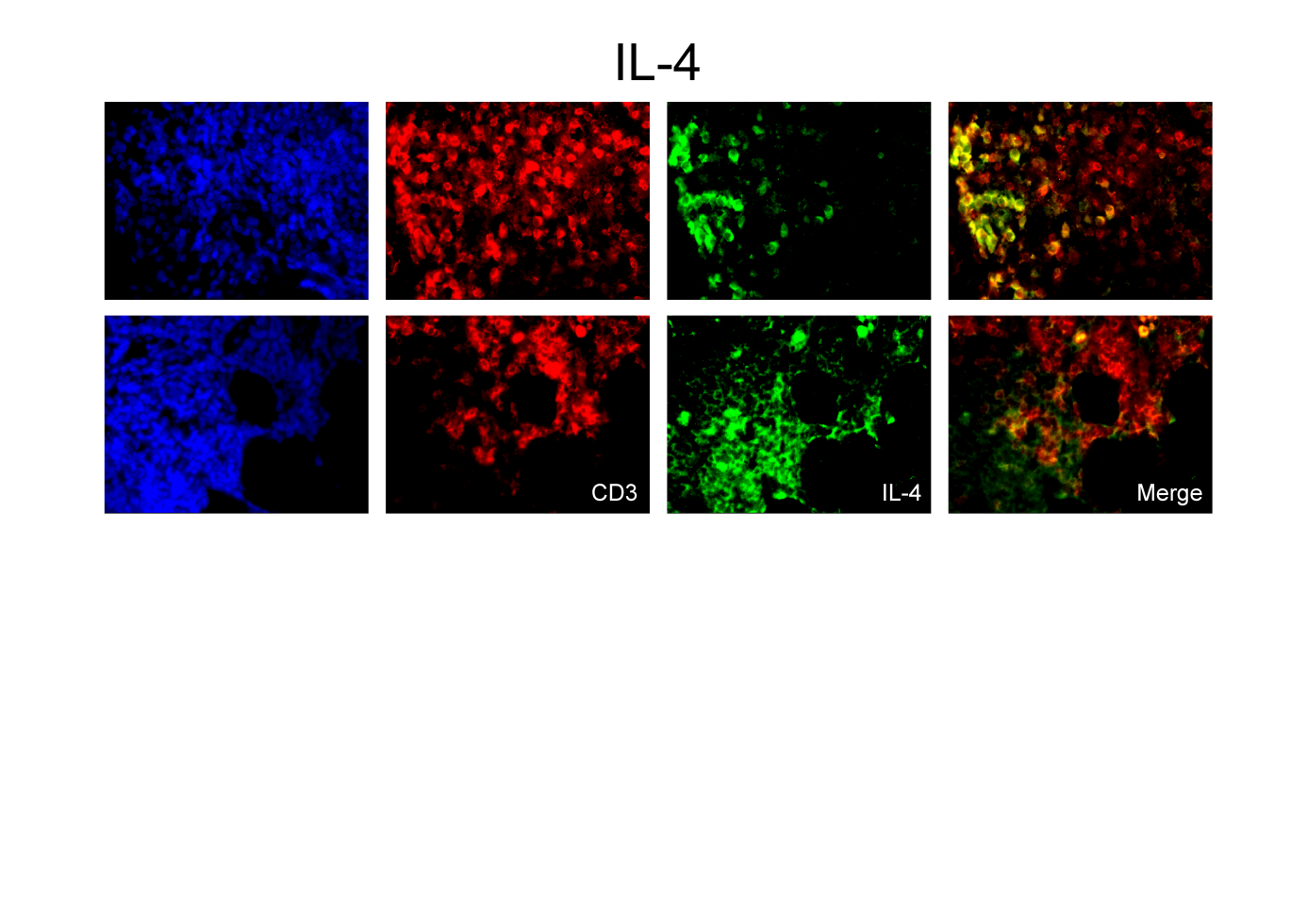
** DAPI CD3 IL-4

**LVV P1**

**LVV P2**

DAPI Merge

**
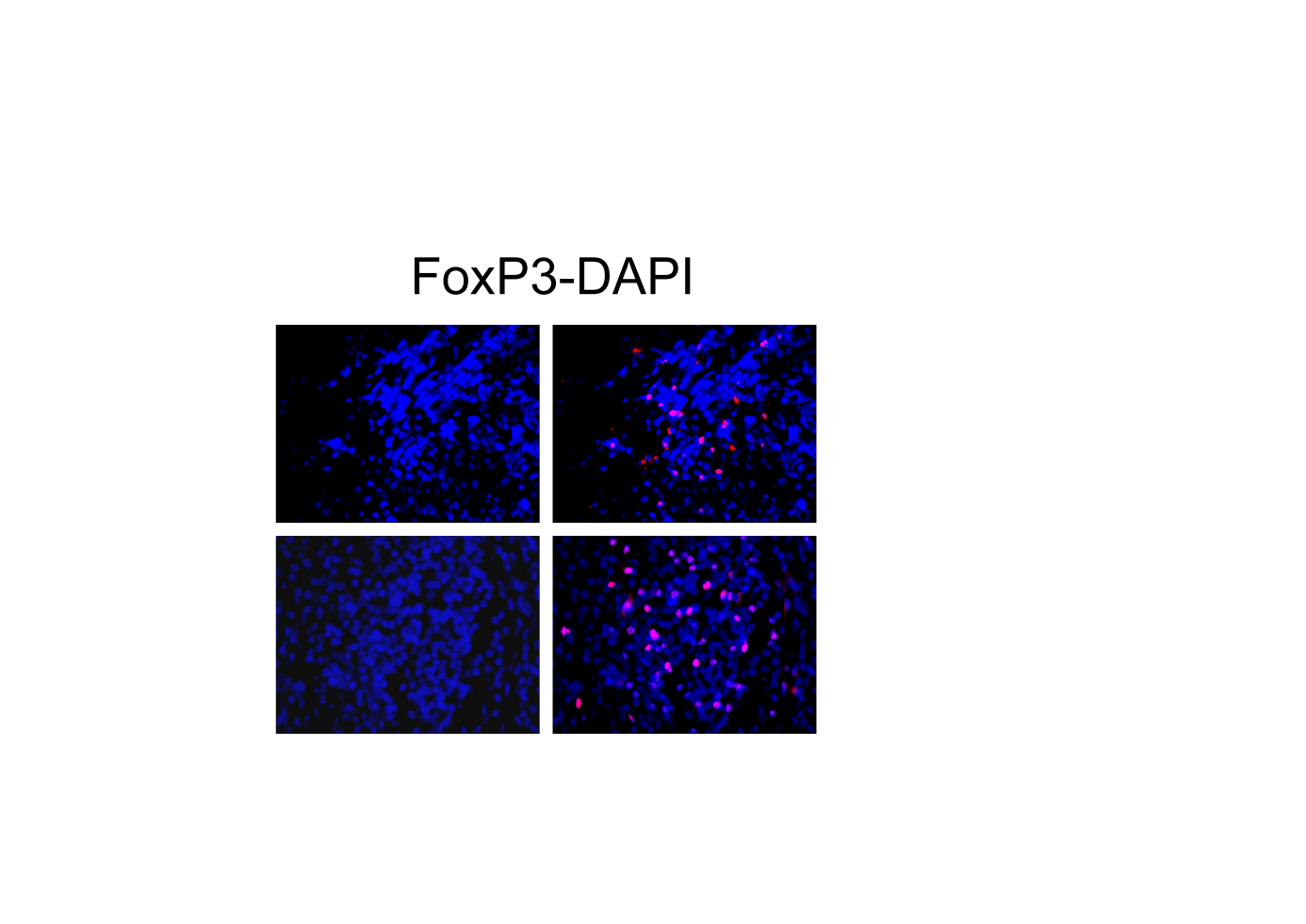
** (FOX and DAPI)

**LVV P1**

**LVV P2**

**Figure S4** : **Gating strategy of intracellular staining of Th1 and Th2 cytokines**
**Figure S5** : **Gating strategy for Treg** **staining**

**Figure S6: Capacity of Treg to prevent the proliferation of Teff (effector T cells). This plot shows the results of 4 experiments.**

Teff + +

Treg - +

**Figure S7** : **Gating strategy for Treg** **in MC and CD4 T cells cocultures**
